# Impact of Electronic Versus Paper Vital Sign Observations on Length of Stay in Trauma Patients: Stepped-Wedge, Cluster Randomized Controlled Trial

**DOI:** 10.2196/10221

**Published:** 2018-10-31

**Authors:** David CW Wong, Julia Knight, Jacqueline Birks, Lionel Tarassenko, Peter J Watkinson

**Affiliations:** 1 Leeds Institute of Health Sciences Faculty of Medicine and Health University of Leeds Leeds United Kingdom; 2 Kadoorie Centre for Critical Care Research and Education Nuffield Department of Clinical Neurosciences University of Oxford Oxford United Kingdom; 3 Centre for Statistics in Medicine University of Oxford Oxford United Kingdom; 4 Institute of Biomedical Engineering Department of Engineering Science University of Oxford Oxford United Kingdom

**Keywords:** vital signs, medical records system, computerized, length of stay, evaluation studies, trauma

## Abstract

**Background:**

Electronic recording of vital sign observations (e-Obs) has become increasingly prevalent in hospital care. The evidence of clinical impact for these systems is mixed.

**Objective:**

The objective of our study was to assess the effect of e-Obs versus paper documentation (paper) on length of stay (time between trauma unit admission and “fit to discharge”) for trauma patients.

**Methods:**

A single-center, randomized stepped-wedge study of e-Obs against paper was conducted in two 26-bed trauma wards at a medium-sized UK teaching hospital. Randomization of the phased intervention order to 12 study areas was computer generated. The primary outcome was length of stay.

**Results:**

A total of 1232 patient episodes were randomized (paper: 628, e-Obs: 604). There were 37 deaths in hospital: 21 in the paper arm and 16 in the e-Obs arm. For discharged patients, the median length of stay was 5.4 (range: 0.2-79.0) days on the paper arm and 5.6 (range: 0.1-236.7) days on the e-Obs arm. Competing risks regression analysis for time to discharge showed no difference between the treatment arms (subhazard ratio: 1.05; 95% CI 0.82-1.35; *P*=.68). A greater proportion of patient episodes contained an Early Warning Score (EWS) ≥3 using the e-Obs system than using paper (subhazard ratio: 1.63; 95% CI 1.28-2.09; *P*<.001). However, there was no difference in the time to the subsequent observation, “escalation time” (hazard ratio 1.05; 95% CI 0.80-1.38; *P*=.70).

**Conclusions:**

The phased introduction of an e-Obs documentation system was not associated with a change in length of stay. A greater proportion of patient episodes contained an EWS≥3 using the e-Obs system, but this was not associated with a change in “escalation time.”

**Trial Registration:**

ISRCTN Registry ISRCTN91040762; http://www.isrctn.com/ISRCTN91040762 (Archived by WebCite at http://www.webcitation.org/72prakGTU)

## Introduction

### Background

Physiological vital signs are regularly measured in hospitalized patients. Deterioration in vital signs often precedes adverse outcomes [1,2]. However, vital sign alterations can go unrecognized, resulting in treatment delay that may worsen outcomes [3-5]. One method for identifying deterioration is the Early Warning Score (EWS), in which a score is given to each set of recorded vital signs. The overall score, the EWS, is the aggregate of scores assigned to each individual vital sign, depending on its level of abnormality. Higher scores indicate greater physiological abnormality [6].

Until recently, vital signs and EWS have been recorded on paper observation charts. The shortcomings of paper charts include incorrect score assignment to the vital signs and incorrect calculation of EWS [7-9], difficulty in interpretation [10], and poor compliance with clinical escalation protocols [11]. Electronic methods for recording vital sign observations and EWS, known as e-Obs, are becoming increasingly common [12-14]. e-Obs systems may circumvent many of these highlighted issues by automatically assigning EWS and prompting appropriate clinical response. The facility to display recent observations and scores on a central station may also improve the ability of clinical staff to recognize patient deterioration. However, the clinical impact of such systems is currently unclear, with studies reporting conflicting results [15,16].

### Objective

In this study, we prospectively assessed whether the deployment of the VitalPAC e-Obs system (VitalPAC; The Learning Clinic), compared with the paper-based system, changed patients’ hospital length of stay (since the conclusion of this study, The Learning Clinic has been acquired by System C, Maidstone, Kent). Our null hypothesis was that length of stay remained unaltered. A cluster-randomized design was not appropriate as the VitalPAC e-Obs system was to be introduced gradually in the trauma unit. We, therefore, evaluated the intervention as it was being introduced using a stepped-wedge study design.

## Methods

### Study Design

We conducted a randomized stepped-wedge interventional study in the two adult inpatient wards of the trauma unit at the John Radcliffe Hospital, Oxford University Hospitals National Health Service Trust. Each ward had 26 inpatient beds. A stepped-wedge study is one in which the intervention is phased into the study population within clusters across successive time periods, with the time being determined by randomization [17]. The full study protocol is available online [18]. Approval for this study was obtained from the National Health Service Research Ethics Committee (REC #11/H0308/11), and the study was registered with ISRCTN (ISRCTN91040762). Informed written consent was sought from all eligible participants after hospital discharge.

### Control and Intervention

Initially, nursing staff measured vital signs (blood pressure, pulse, oxygen saturation, temperature, respiratory rate, and consciousness level) using spot-check monitors and documented the result on paper. Nurses manually calculated the EWS and recorded the score on paper. The EWS used was the previously published centile-EWS [19], in which a score of 3 or more (EWS≥3) requires urgent intervention.

The intervention was the VitalPAC e-Obs system, which allowed vital signs to be documented on a hand-held device. The EWS was then automatically calculated, and relevant hospital guidance for escalation was displayed. EWS were also displayed at a central patient ward list that adjusted colors and symbols to prompt nurses to record timely observations according to the hospital protocol.

Prior to study commencement, VitalPAC was installed and tested and staff were trained to use the system. Refresher teaching was also provided for the paper system, and staff were reminded of local clinical escalation policy. During the training period, the paper system was used throughout the trauma unit, but staff had the opportunity to enter data into a test installation of VitalPAC. Study staff attended the ward daily during the week and once at weekends, providing top-up training throughout the duration of the study.

### Trial Design

The e-Obs intervention was phased in 12 clusters. Each cluster was a physical zone within the trauma unit consisting of a 4-bed bay or a collection of six 1-bed side rooms. The clusters received the electronic intervention sequentially. A new zone switched from the control to the intervention every 3 weeks on Tuesday at 2 pm.

### Participants

All episodes from patients aged ≥16 years admitted to the trauma unit during the study period were considered for eligibility. Episodes were excluded from analysis if the treatment plan for the patient was palliative at admission to the ward.

### Study Data

Clinical and demographic data on study participants were obtained from an electronic patient record (*Casenotes*) and from paper medical records. Research nurses collated the following: age, sex, ethnicity, American Society of Anesthesiologists score, reason for admission, and admission method (emergency, elective, between wards) for each study participant.

We also recorded initial ward and zone on the trauma unit; date and time (hh:mm) of admission into the ward; date, time (hh:mm), and clinician-recorded EWS for the first vital sign observations recorded on the trauma unit; total number of vital sign observations on the unit recorded electronically and on paper; “fit to discharge” date; actual hospital discharge date and time (hh:mm); in-hospital mortality and 30-day mortality following ward admission; and unplanned admission to the intensive care unit (ICU) or a cardiac arrest for each episode.

Two other electronic sources, *Cerner Millenium* (Cerner, Kansas City, MO) and *Bluespier* (Bluespier International, Worcestershire), were used to validate the information. Mortality status at 30 days after admission was verified using data from the National Health Service personal demographic service.

### Outcomes

The primary endpoint was length of stay (the time from admission to the trauma unit until “fit to discharge”). “Fit to discharge” was defined as the first of the following: discharged from the ward to home or alternative care or accepted by social services as a “delayed discharge.” This outcome measure was chosen because some trauma patients were known to have extended stays while waiting for suitable support mechanisms to be put in place outside hospital. Secondary endpoints were mortality (in-hospital and 30-day following ward admission), whether a patient experienced a cardiac arrest or an unplanned ICU admission; the time between observations (length of stay/total number of observations); the time until a patient first scored EWS≥3; and the time between the first observation that scored EWS≥3 and the subsequent observation (“escalation time”).

### Sample Size

The number of participants was determined by the speed at which zones transitioned from paper to e-Obs. Slower transitions would include more participants for each step, thereby increasing the power of the study. However, clinical staff wished to minimize the concurrent use of multiple systems. Therefore, a clinically accepted transition rate of 1 zone every 3 weeks was chosen, and no sample size calculation was undertaken.

### Randomization

A random sequence generated using MATLAB (function *randperm* [20]) determined the order in which zones received e-Obs.

All zones were recruited and enrolled at baseline and followed for the entire duration of the study. Research nurses administered the sequential assignment to the e-Obs intervention. The nurses visited the ward during each transition to facilitate adherence to the change from paper to e-Obs.

Patients were allocated to either paper or electronic recording of observations based on the zone to which they were allocated on arrival to the trauma unit. Patients remained with the same recording method even if subsequently moved. Allocation to the initial zone was determined by normal ward practices, which remained unchanged during the study period. Therefore, the allocation ratio could not be determined a priori.

### Blinding

The randomization sequence was concealed from patients, clinical staff, and all researchers involved in data collection until the day a new zone was due to receive e-Obs. It was not possible to conceal the intervention.

### Statistical Methods

Episode characteristics, including patient age and length of stay, were summarized by study arm. A time-to-event analysis, Cox proportional hazards regression, was undertaken for length of stay with the competing risk of death in hospital, for death in hospital with discharge from hospital as a competing risk, and for time to first EWS≥3. The intervention arm, step, age, and sex of patients were included as covariates. Subhazard ratios were calculated with respect to paper charts, age>80 years, and male sex. The step was included as a continuous measure of time and as a factor with 13 levels. The study design has two levels, patient and study zone. The SEs of the coefficients in the Cox proportional hazards model were adjusted for the cluster variable, study zone. A similar competing risks Cox regression analysis was undertaken for “escalation time,” censored at 200 hours.

The binary outcome, death within 30 days of admission, was analyzed using a logistic regression model and the SEs of the coefficients were adjusted for the cluster variable, study zone. The numbers of cardiac arrests and admissions to ICU were reported.

All analyses were performed on an intention-to-treat basis, with patients analyzed according to the randomization intervention. Furthermore, all analyses were completed for hospital episodes. A patient may have had multiple episodes consisting of distinct admissions to the study wards for unrelated reasons. We assumed a priori that the number of such admissions was small such that each episode may be treated as an independent event. Post hoc, we repeated the analyses on the per-protocol populations.

All statistical analyses were performed using Stata (Stata Statistical Software: Release 14. College Station, TX; StataCorp LP) [21].

## Results

**Figure 1:**
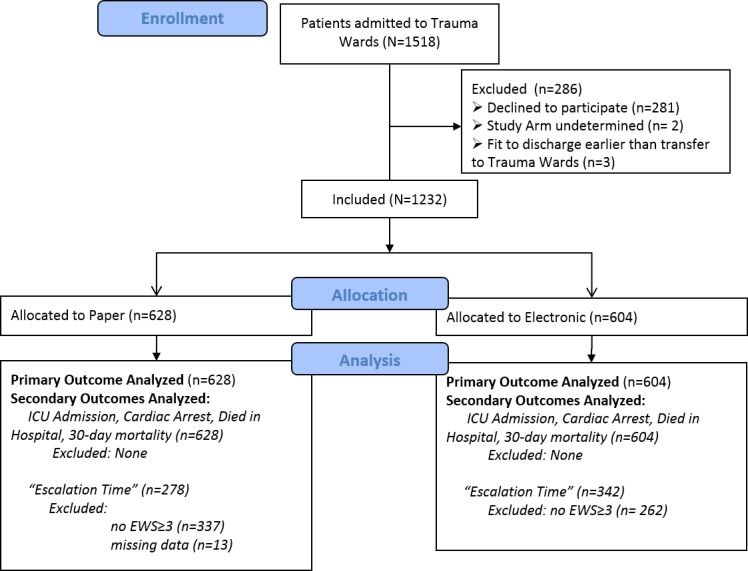
Flow chart for patients’ enrollment in the study. “Escalation Time” is the time between the first observation that scored Early Warning Score (EWS)≥3 and the subsequent observation. ICU: intensive care unit.

A total of 1518 admissions to the trauma unit were recorded between August 31, 2011 and May 31, 2012. After excluding 286 episodes that did not meet the study criteria or for which patients had declined consent, 1232 episodes (from 1199 patients) were included for analysis (Figure 1).

Of the included episodes, 628 were randomized to paper and 604 to e-Obs. Moreover, 873 episodes (paper: 558, e-Obs: 315) had vital sign observations that were fully consistent with the randomized intervention. A further 194 episodes (paper: 32, e-Obs: 162) had over 80% of observations on the allocated intervention. Vital sign observation charts were absent from the paper notes for 13 episodes.

Baseline characteristics of the study patients are shown in Table 1. Allocation between the study arms was almost equal (paper: 628/1232, 50.97%). There were no significant differences in any of the measured characteristics. In all analyses, the intraclass correlation coefficient for patients within a study area was not significantly different from zero.

There were 37 deaths in hospital: 21 in the paper arm and 16 in the e-Obs arm. For patients who were discharged, the median length of stay (time from admission to “fit to discharge”) was 5.4 (range: 0.2-79.0) days on the paper arm (607 patients) and 5.6 (range: 0.1-236.7) days on the e-Obs arm (588 patients). Longer time to discharge was associated with greater age, but there was no difference between the treatment arms (Table 2).

Results from the competing risks Cox regression analysis for time to death in hospital with discharge from hospital as a competing risk are reported in Table 2. There was no difference between the treatment arms.

There were 45 deaths within 30 days from admission to hospital: 23 in the paper arm and 22 in the e-Obs arm. The results from the logistic regression analysis of the number of deaths within 30 days of admission are reported in Table 2. There was no difference between the treatment arms.

There were 8 admissions to the ICU from the paper arm and 7 from the e-Obs arm, and there were 2 cardiac arrests in each arm. The median time between observations for those without a delayed discharge was 7.1 (interquartile range [IQR]: 5.0-9.8) hours on paper and 7.0 (IQR: 5.3-9.5) hours on e-Obs.

**Figure 2:**
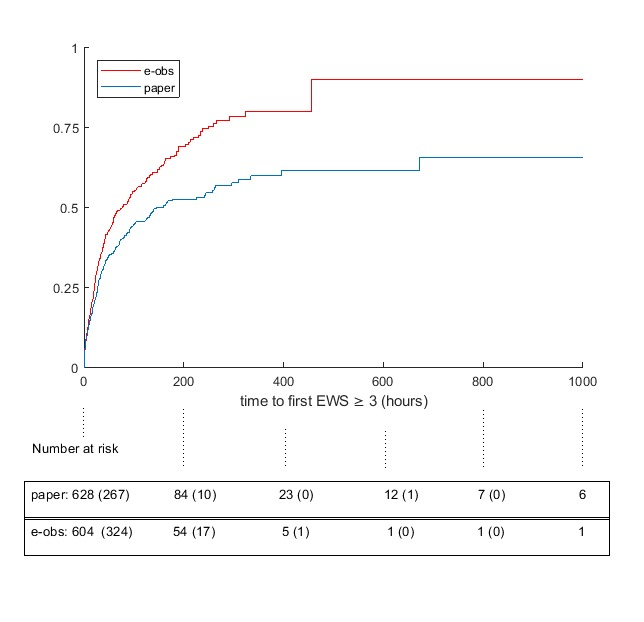
Kaplan-Meier failure estimates for time (hours) from admission until first Early Warning Score (EWS)≥3. e-Obs: electronic recording of vital sign observations.

Figure 2 shows the Kaplan-Meier estimates of time to first EWS≥3. At least one EWS≥3 was recorded for 278 and 342 episodes in the paper and e-Obs arms, respectively. The number at risk indicates the episodes that had not had an EWS≥3 after {0, 200, 400, 600, 800, and 1000} hours. Numbers in parentheses are those who had an EWS≥3 before the next time point. Moreover, 7 episodes in the e-Obs arm had no recorded observations, while 13 episodes in the paper arm had missing data (observation chart missing from notes). The subhazard ratio for e-Obs with respect to paper from a competing risks Cox regression analysis for time to first EWS ≥3 with death in hospital as a competing risk including age>80 years, step, and sex was 1.63 (95% CI 1.28-2.09; *P*<.001).

**Figure 3:**
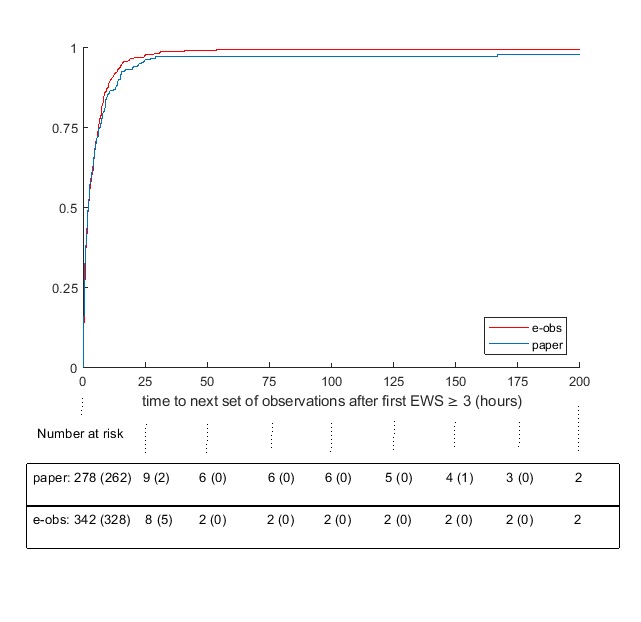
Kaplan-Meier failure estimates for time (hours) to next set of observations after the first Early Warning Score (EWS)≥3. e-Obs: electronic recording of vital sign observations.

Figure 3 shows the Kaplan-Meier estimates of *escalation time*. Inclusion in the analysis was conditional on reaching an EWS of ≥3. The number at risk indicates the episodes that had not yet had an observation after {0, 25, 50,..., 200} hours. Numbers in parentheses are those who had the next set of observations before the next time point. Patients could be censored at any time due to discharge from hospital or death in hospital. Three episodes had the next observation after more than 50 hours, 18 were discharged from hospital, and 3 died in the hospital before a further set of observations. The hazard ratio for e-Obs with respect to paper from a competing risk Cox regression analysis for time to escalation including age>80 years, step, and sex was 1.05 (95% CI 0.80-1.38; *P*=.70).

All results presented here are for intention-to-treat analysis. The results of the per-protocol analysis are shown in Multimedia Appendix 1. On per-protocol analysis, we found no difference in “escalation time” or length of stay. Per-protocol in-hospital time to mortality analysis suggested a mortality benefit in favor of e-Obs, but this was not sustained at 30 days.

**Table 1:** Patient baseline characteristics (N=1232 episodes).

Characteristic		Paper (n=628)	e-Obs^a^ (n=604)
Age (years) mean (SD)		58.1 (23.4)	60.4 (23.3)
Males, n (%)		308 (49.0)	316 (52.3)
Ward 1:Ward 2		312:316	280:324
**Ethnicity, n (%)**			
	White British	471 (74.9)	446 (73.6)
	Not stated	110 (17.5)	129 (21.3)
	Other	48 (7.6)	31 (5.1)
**Reason for admission: injury type, n (%)**			
	Lower limb (excluding neck of femur)	179 (28.5)	188 (31.1)
	Neck of femur	160 (25.5)	169 (28.0)
	Upper limb (excluding wrist)	59 (9.4)	63 (10.4)
	Polytrauma (excluding head)	48 (7.6)	45 (7.5)
	Wrist	38 (6.1)	21 (3.5)
	Spinal trauma	33 (5.3)	30 (5.0)
	Polytrauma + head	29 (4.6)	23 (3.8)
	Nontrauma	29 (4.6)	15 (2.5)
	Other	53 (8.4)	50 (8.3)
**Primary specialty, n (%)**			
	Trauma	581 (92.5)	574 (95.0)
	Other	47 (7.5)	30 (4.9)
**Admission method, n (%)**			
	Emergency department (not via intensive care unit)	323 (51.4)	342 (56.3)
	Trauma clinic within Oxford University Hospitals Trust	108 (17.2)	107 (17.6)
	Other ward	89 (14.3)	57 (9.7)
	Transfer from other United Kingdom hospital	24 (3.8)	24 (4.0)
	Emergency department via emergency admissions unit	50 (7.9)	50 (8.2)
	Other	34 (5.4)	24 (4.0)
**American Society of Anesthesiologists score, n (%)**			
	1	101(16.1)	97 (16.1)
	2	97 (15.4)	107 (17.7)
	3	92 (14.6)	86 (14.2)
	4	18 (2.9)	22 (3.7)
	5	3 (0.5)	1 (0.2)
	1E-5E	11 (1.8)	12(2.0)
	Unrecorded	180 (28.7)	169 (28.0)
	Not applicable	126 (20.0)	110 (18.2)

^a^e-Obs: electronic recording of vital sign observations.

**Table 2:** Results from the competing risks regression analysis for time to discharge from hospital with death in hospital as a competing risk, from the competing risks regression analysis for time to death in hospital with discharge from hospital as a competing risk, and the logistic regression for number of deaths within 30 days from admission.

Variable	Time to discharge from hospital with death in hospital as a competing risk		Time to death in hospital with discharge from hospital as a competing risk		Number of deaths within 30 days from admission	
	Subhazard ratio (95% CI)	*P* value	Subhazard ratio (95% CI)	*P* value	Odds ratio (95% CI)	*P* value
e-Obs^a^	1.05 (0.82-1.35)	.68	0.77 (0.42-1.40)	.39	0.82 (0.46-1.47)	.51
Step	1.00 (0.97-1.03)	.99	0.98 (0.88-1.10)	.72	1.01 (0.92-1.12)	.80
Age ≥80 years	0.62 (0.57-0.67)	<.001	5.69 (3.48-9.30)	<.001	8.93 (5.69-14.01)	<.001
Female	1.09 (0.96-1.25)	.19	0.63 (0.35-1.12)	.11	0.60 (0.39-0.94)	.02

^a^e-Obs: electronic recording of vital sign observations.

## Discussion

### Principal Findings

To our knowledge, this is the first stepped-wedge evaluation of an e-Obs intervention. We found no difference in length of stay (the primary outcome) between paper and e-Obs. A significantly greater proportion of patient episodes contained an EWS≥3 using the e-Obs system than using paper. However, we found no difference in “escalation time.” Per-protocol analysis showed improved in-hospital survival with e-Obs, but this was not shown either on intention-to-treat analysis or for outcome at 30 days. The absence of intraclass correlation indicates that patient groups were well matched at each individual step level.

### Limitations

Although 86.61% (1067/1232) of patients had over 80% of their observations in the correct format, cross over was unequal. Only 79.0% (477/604) of patients in the electronic observations arm had more than 80% of their observations recorded electronically. The presence of multiple documentation systems in the wards may have led to confusion and suboptimal system use. To avoid this issue, future studies might ensure clear physical separation between zones to prevent study arm contamination. Our follow-up study over 4 hospitals instead considers whole wards as a study zone [22].

Length of stay is a complex outcome, risking confounding by competing interventions. However, it is an important outcome for patients and their families [23], as well as clinicians and managers, and has been recommended as a potential outcome measure within the National Institute for Health and Care Excellence recommendations for recognizing patient deterioration in hospital [24]. We used it as our primary outcome as delayed detection of deterioration might be expected to increase length of stay even where the delay or deterioration was not sufficient to warrant ICU admission or cause death. Indeed, improved recognition of clinical deterioration is associated with earlier discharge [25] and has previously been used in observational studies assessing e-Obs systems [13,15,26]. To minimize the effect of competing interventions, we used “fit to discharge” rather than actual discharge time and took account of the competing risk of death in our analysis. We included other outcome measures that would be expected to be associated with more extreme delays in recognizing deterioration (death and ICU admission) as secondary outcome measures.

### Interpretation

In this study, more patient episodes contained an EWS≥3 using the e-Obs system than paper. We have previously shown paper documentation errors to be biased toward values lower than the true EWS, particularly when a patient first develops physiological instability [27]. As the groups were otherwise well balanced, it is likely that the result is explained by a bias in paper documentation. As the clinical behavior underlying this bias appears to be related to the actual instability of the patient, it is unclear whether removing the bias will affect patient outcomes [27].

Despite differences in documented EWS, there was no difference in the timeliness of observations between the two cohorts. This supports the results reported by Hands et al, in which an e-Obs system was introduced to all adult inpatient areas of one hospital [11]. The time of vital sign observations was recorded. They found that observations were more frequently recorded at particular hours in the day, rather than simply responding to escalation algorithms.

There was also no difference in the primary outcome, patient length of stay from admission to the trauma unit until “fit to discharge” after accounting for potential confounding variables. Previous evaluations of e-Obs systems have focused on process improvements such as data accuracy and speed of documentation [7,28-30]. More recently, longitudinal data have been used to assess the impact of e-Obs on patient mortality via before-and-after analysis, with conflicting results [15,16]. Dawes et al found a 2-day reduction in average length of stay after introduction of the VitalPAC e-Obs system when comparing results in 2010 to those in 2005 [15]. However, further exploration suggested this finding was due to a decreased severity of admission rather than a change in in-hospital care. Jones et al reported reductions in length of stay using the Patientrack e-Obs system [13]. Subsequent correspondence suggested that this may have resulted from changes in discharge processes rather than changes in care resulting from the e-Obs system [31,32].

A more recent study suggested substantial reductions in hospital mortality in two hospitals that implemented VitalPAC [16]. Although our per-protocol analysis also showed a survival benefit with e-Obs, this was a post hoc analysis of a secondary outcome and was not found in either intention-to-treat analysis or 30-day mortality (analyzing either intention-to-treat or per-protocol groups). We, therefore, do not think great weight should be attached to this outcome, particularly as we did not find any change in observation frequency in those becoming unstable to support the hypothesis that e-Obs caused a change in care.

The previous before-and-after studies are inherently limited in their ability to account for temporal changes in covariates [33]. Our stepped-wedge design and relatively short total study time reduced the risk of other major changes in practice affecting our outcomes. The stepped-wedge methodology employed here is a practical choice for phased interventions that allows for control of temporal covariates because both control and intervention are active over the whole study period [34]. One disadvantage of the stepped-wedge design is that there is no established consensus on the most appropriate methods of modeling the data [35]. However, if properly analyzed, the quality of evidence is better than that of before-and-after studies and approaches that of randomized controlled trials [36].

Although e-Obs had no effect on patient outcomes in our study, there are some positive findings. First, the frequency of observations before and after e-Obs remained stable. Furthermore, although the timeliness of observations when patients were physiologically unstable did not improve, they also did not worsen, matching previous reports of e-Obs introduction [13]. These findings suggest that it is possible to introduce an e-Obs system without adversely affecting these ward staff functions. Without an adverse effect, the availability of the vital signs electronically brings the possibility of benefits outside the patients studied, or in the future that may make e-Obs worthwhile.

### Generalizability

The results here are specific to the VitalPAC e-Obs system. This system contains some key features that are available in alternative e-Obs solutions; these include automatic EWS calculation and real-time ward lists [12]. The results are also location specific. While trauma was chosen as a representative specialty that contained a wide range of care, results may not be true in other hospital contexts. Although these factors may reduce the generalizability of results, the results are robust due to the large number of study participants and the stepped-wedge study design.

### Conclusions

The introduction of an electronic system for recording vital sign observations was not associated with reduction in time from admission to the trauma unit until “fit to discharge.” More patient episodes contained an EWS≥3 using the e-Obs system, but this was not associated with a change in “escalation time.”

## Appendix

Multimedia Appendix 1 Per-protocol analysis.

Multimedia Appendix 2 CONSORT‐EHEALTH checklist (V 1.6.1).

## Abbreviations

e-Obs: electronic recording of vital sign observations
EWS: Early Warning Score
ICU: intensive care unit
