# Supplementary material for: Impact of Electronic Versus Paper Vital Sign Observations on Length of Stay in Trauma Patients: Stepped-Wedge, Cluster Randomized Controlled Trial
Source: JMIR Med Inform. 2018 Oct 31;6(4):e10221. doi: 10.2196/10221 (PMC6236204; doi:10.2196/10221)
Supplement: Multimedia Appendix 1 [file medinform_v6i4e10221_app1.pdf]

Multimedia Appendix 1. Per-protocol analysis

There were 23 deaths in hospital, 19 in the paper arm and 4 in the e-Obs arm. For the patients who were discharged the median length of stay (time from admission to “fit to discharge”) was 5.4 days (range: 0.2 to 79.0) and 5.1 days (range: 0.1 to 91) on the paper (551 patients) and e-Obs arm (318 patients), respectively. Longer time to discharge was associated with greater age, but there was no difference between the treatment arms (Table 1).

The results from the competing risks Cox regression analysis for time to death in hospital with discharge from hospital as a competing risk are reported in Table 1. There was a statistically significant difference between the treatment arms in favor of longer survival in patients documented using e-Obs.

There were 30 deaths within 30 days from admission to hospital, 21 in the paper arm and 9 in the e-Obs arm. The results from the logistic regression analysis of the numbers of deaths within 30 days of admission are reported in Table 1. There was no difference between the treatment arms.

Table 1. Results from the competing risks regression analysis for time to discharge from hospital with death in hospital as a competing risk, from the competing risks regression analysis for time to death in hospital with discharge from hospital as a competing risk, and the logistic regression for number of deaths within 30 days from admission.

100% compliance (n=890)	Time to discharge from hospital with death in hospital as a competing risk	Time to death in hospital with discharge from hospital as a competing risk	Number of deaths within 30 days from admission
Variable	Subhazard ratio (95% CI) *P* value	Subhazard ratio (95% CI) *P* value	Odds ratio (95% CI) *P* value
e-Obs	1.02 (0.78-1.33) *P*=.89	0.32 (0.13-0.78) *P*=.01	0.51 (0.19-1.36) *P*=.18
Step	1.02 (0.99-1.05) *P*=.15	1.00 (0.89-1.12) *P*=.98	1.04 (0.94-1.16) *P*=.44
Age≥80	0.56 (0.52-0.62) *P*<.001	9.76 (5.28-18.00) *P*<.001	14.65 (7.71-27.86) *P*<.001
Female	1.17 (1.04-1.31) *P*=.01	0.45 (0.24-0.84) *P*=.01	0.44 (0.27-0.70) *P*=0.001

There were 6 admissions to the ICU from the paper arm and 0 from the e-Obs arm, as well as 2 cardiac arrests from the paper arm and 0 from the e-Obs arm. There were 2 missing values in the paper arm for admission to ICU and cardiac arrest.

Figure 1. Kaplan-Meier failure estimates for time (hours) from admission until first EWS≥ 3. The number at risk indicates the episodes that had not had an EWS≥ 3 after {0, 200, 400, 600, 800, and 1000} hours. Numbers in parentheses are those who had an EWS≥3 before the next time point.

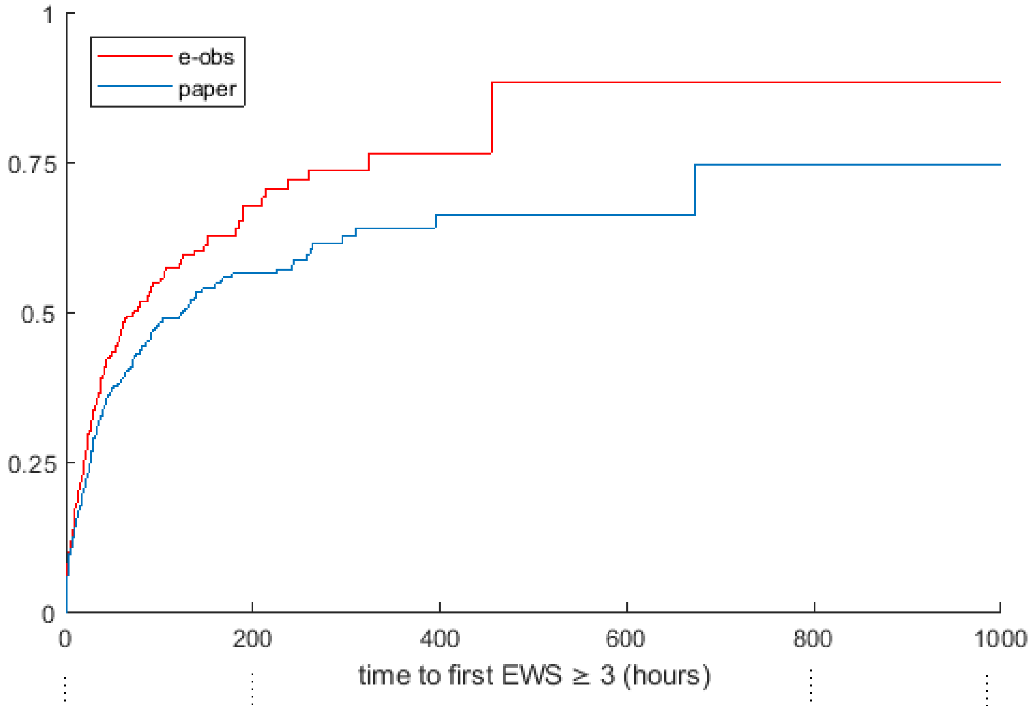

Number at risk

paper: 570(258)	65(9)	15(0)	6(1)	3(0)	2
e-obs: 322(258)	26(9)	4(0)	1(0)	1(0)	1

Figure 1 shows the Kaplan-Meier estimates of time to first EWS≥3. The subhazard ratio for e-Obs with respect to the paper arm from a competing risks Cox regression for time to first EWS ≥3 including age>80, step, and sex was 1.38 (95% CI 1.03-1.83; *P*=.03).

Figure 2 shows the Kaplan-Meier estimates of "escalation time" to the subsequent observation time. Accordingly, 268 and 178 patients in the paper arm and e-Obs arm had an EWS≥3, respectively. Data were censored at an "escalation time" of 200 hours. Moreover, 2 episodes had an observation after more than 50 hours, 17 were discharged from hospital, and 1 patient died in the hospital before a further set of observations. The subhazard ratio for e-Obs with respect to paper from a Cox regression for time to escalation including age>80, step, and sex was 1.19 (95% CI 0.84-1.69; *P*=.33).

Figure 2. Kaplan-Meier failure estimates for time (hours) to next set of observations after the first EWS≥3. The analysis is conditional on reaching an EWS≥3. The number at risk indicates the episodes that did not have an observation after {0, 25, 50,..., 200} hours. Numbers in parentheses are those who were censored at the corresponding times.

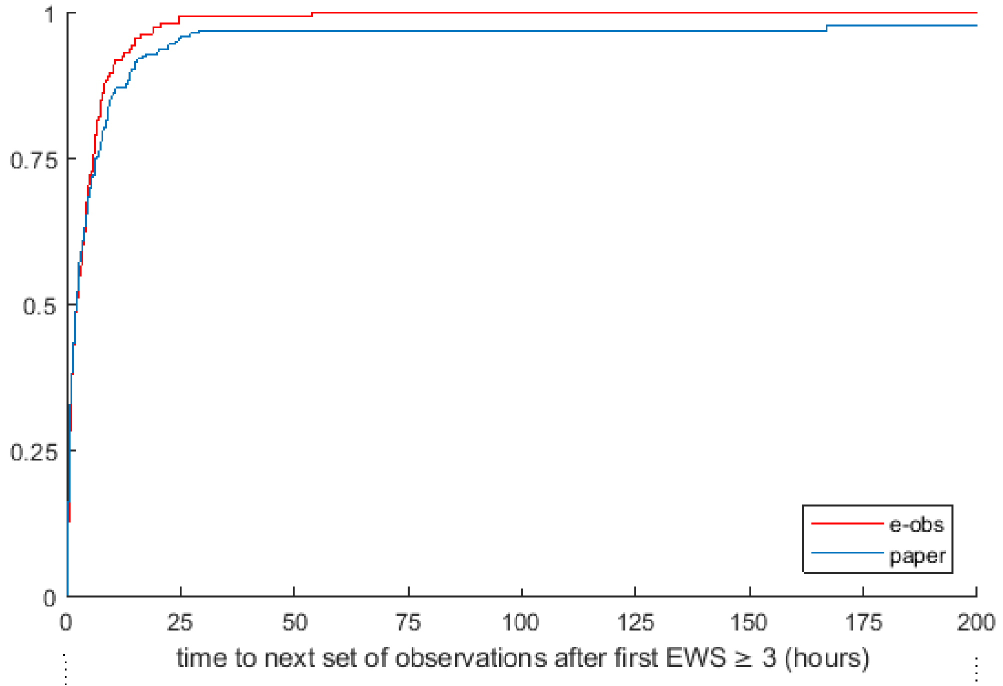

Number at risk

paper: 268(252)	9(2)	6(0)	6(0)	6(0)	5(0)	4(1)	3(0)	2
paper: 178(173)	1(0)	1(0)	0(0)	0(0)	0(0)	0(0)	0(0)	0
